# Supplementary material for: COVID-19 Virus and Vaccination Attitudes among Healthcare Workers in Michigan: A Cross-Sectional Study
Source: Vaccines (Basel). 2023 Jun 16;11(6):1105. doi: 10.3390/vaccines11061105 (PMC10304020; doi:10.3390/vaccines11061105)
Supplement: Supplementary file 1 [file vaccines-11-01105-s001.zip › vaccines-2418694-supplementary.pdf]

Supplementary FIle

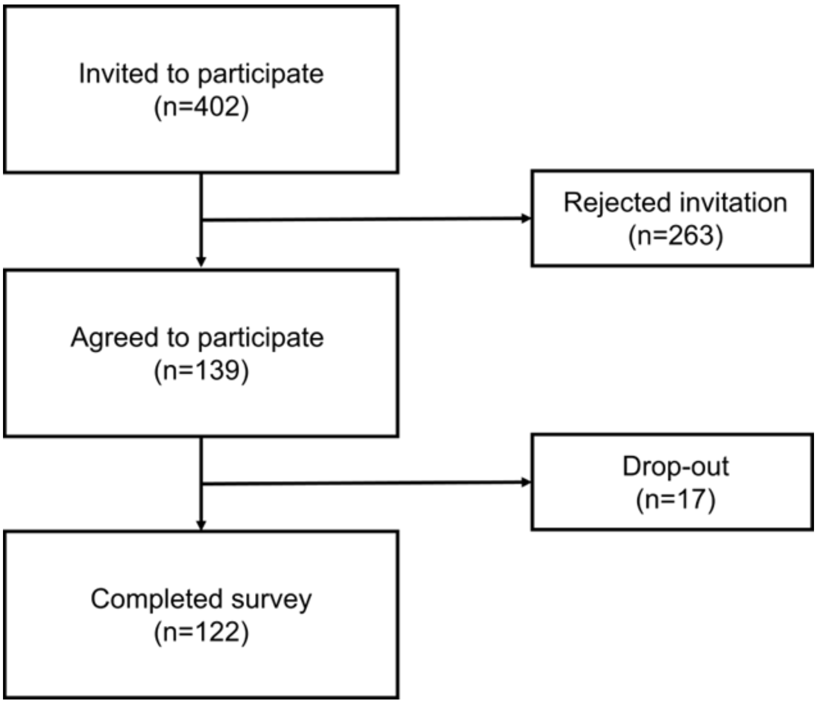

Figure S1. Response rate. 402 clinicians were invited to participate in the survey. 34.5% (n=139) accepted the invitation. 30.3% accepted the invitation and completed the survey.

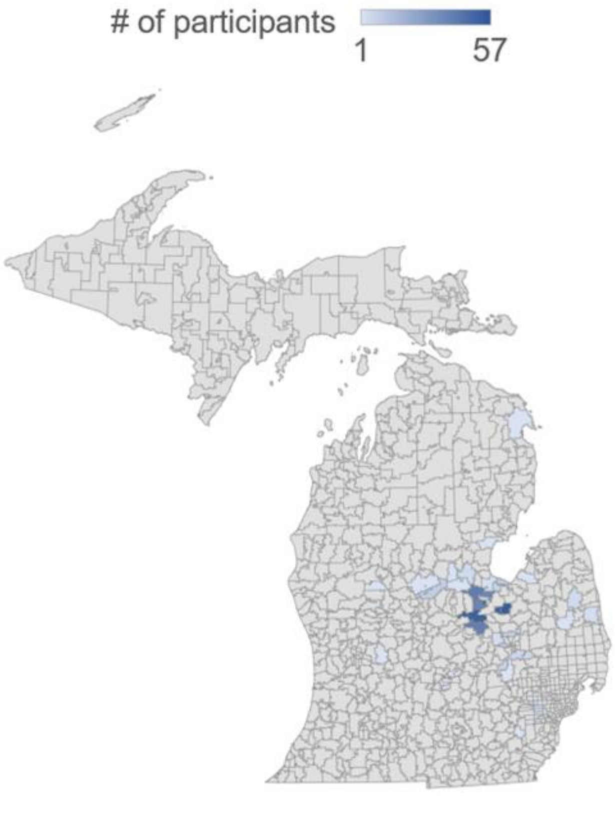

Figure S2. Heat map of participant residence. Residence of clinician participation on Michigan state map.

Supplemental documents

Document S1. 45-item questionnaire given to HCWs in this study.

Supplemental Document S1: 45-item questionnaire given to HCWs in this study.

DEMO1 How would you describe your gender?

- ○ Female
- ○ Male
- ○ Transgender Female
- ○ Transgender Male
- ○ Gender Variant/Non-Conforming
- ○ Other (please specify): ____
- ○ Prefer Not to Answer

DEMO2 What is your age?

- ○ 18 – 24 years old
- ○ 25 – 34 years old
- ○ 35 – 44 years old
- ○ 45 – 54 years old
- ○ 55 – 64 years old
- ○ 65+ years old

DEMO3 Please specify your race.

- ○ White or Caucasian
- ○ Black or African American
- ○ American Indian or Alaska Native
- ○ Asian
- ○ Native Hawaiian or Other Pacific Islander
- ○ Other (please specify): ____

DEMO4
Please specify your ethnicity.

- ○ Hispanic
- ○ Non-Hispanic

DEMO5a Current Residence: City/Town Name

____

DEMO5b Current Residence: Zip Code

____

DEMO6 Provider type:

- ○ Nurse
- ○ Nurse Practitioner
- ○ Pharmacist
- ○ Physician Assistant
- ○ Physician
- ○ Other ______________________________

DEMO7 Professional training status:

- ○ Licensed Provider
- ○ Resident/Fellow
- ○ Intern

DEMO8 What is your medical specialty?
Select <u>all that apply</u>.

- ☐ Allergy and Immunology
- ☐ Anatomic/Clinical Pathology
- ☐ Anesthesiology
- ☐ Cardiovascular Disease
- ☐ Child and Adolescent Psychiatry
- ☐ Critical Care Medicine
- ☐ Dermatology
- ☐ Emergency Medicine
- ☐ Endocrinology, Diabetes and Metabolism
- ☐ Family Medicine/General Practice
- ☐ Gastroenterology
- ☐ General Surgery
- ☐ Geriatric Medicine
- ☐ Hematology and Oncology
- ☐ Infectious Disease
- ☐ Internal Medicine
- ☐ Internal Medicine/Pediatrics

- [ ] Interventional Cardiology
- [ ] Neonatal-Perinatal Medicine
- [ ] Nephrology
- [ ] Neurological Surgery
- [ ] Neurology
- [ ] Neuroradiology
- [ ] Obstetrics and Gynecology
- [ ] Ophthalmology
- [ ] Orthopedic Surgery
- [ ] Otolaryngology
- [ ] Pain Medicine and Pain Management
- [ ] Pediatric Cardiology
- [ ] Pediatric Hematology/Oncology
- [ ] Pediatrics
- [ ] Physical Medicine and Rehabilitation
- [ ] Plastic Surgery
- [ ] Preventive Medicine
- [ ] Psychiatry

- [ ] Pulmonary Disease
- [ ] Radiation Oncology
- [ ] Radiology and Diagnostic Radiology
- [ ] Rheumatology
- [ ] Sports Medicine (Orthopedic Surgery)
- [ ] Thoracic Surgery
- [ ] Urology
- [ ] Vascular and Interventional Radiology
- [ ] Vascular Surgery
- [ ] Other (please specify): ____________________

FLU **The following questions ask about your opinion on seasonal flu vaccinations.**

FLU1 Have you been vaccinated against influenza (the flu) in the **last year**?

- ○ Yes
- ○ No
- ○ Don't know

FLU2 Do you plan on getting this season’s influenza vaccine **this fall**?

- ○ Yes, I am planning on receiving the vaccine.
- ○ No, I am not planning on receiving the vaccine.
- ○ Undecided

COVID **The following ask about your COVID-19 infection and vaccine status.**

COVID1 Since January 2020, have you been <u>tested</u> for the COVID-19 virus?

- ○ Yes
- ○ No
- ○ Don’t know

COVID2 If given the opportunity to take a COVID-19 vaccine, how likely is it that you would get the vaccine/shot?

- ○ Definitely will not
- ○ Very unlikely
- ○ Somewhat unlikely
- ○ Somewhat likely
- ○ Very likely
- ○ Definitely will
- ○ I have already received the COVID-19 vaccine

COVID3 Have you received a COVID-19 vaccine?

- ○ Yes, I have received the first dose of a two dose COVID-19 vaccine (i.e. Pfizer-BioNTech, Moderna).
- ○ Yes, I have received both doses of a two dose COVID-19 vaccine (i.e. Pfizer-BioNTech, Moderna).
- ○ Yes, I have received one dose of a one dose COVID-19 vaccine (i.e. Johnson & Johnson’s Janssen).
- ○ I am planning on getting vaccinated but have not received it yet.
- ○ I am NOT planning on getting vaccinated.

COVID_Vac **The following questions ask about your opinion on the COVID-19 vaccine.**

COVID_Vac_op Please select '**AGREE**,' '**DISAGREE**,' or '**UNSURE'** for the following statements.

	AGREE	DISAGREE	UNSUR
I am concerned that I will be infected by the COVID-19 virus.	○	○	○
I believe that CDC recommendations, such as wearing a mask and social distancing, are effective against a COVID-19 infection.	○	○	○
Even if I get infected with COVID-19, I believe that I am healthy enough to fight it off.	○	○	○
I am concerned about the mild reactions of a COVID-19 infection.	○	○	○
I am concerned about the severe reactions of a COVID-19 infection.	○	○	○
I trust that a COVID-19 vaccine will give me immunity against a COVID-19 infection because it is safe and effective against a COVID-19 infection.	○	○	○
I believe that it will benefit my body if I take a COVID-19 vaccine.	○	○	○
I have been offered a COVID-19 vaccine.	○	○	○
I believe that the COVID-19 vaccines have adequate testing and results.	○	○	○

The COVID-19 vaccines were developed and tested too quickly.	○	○	○
I am concerned about the side effects of a COVID-19 vaccine.	○	○	○
I am concerned about the long-term side effects of a COVID-19 vaccine.	○	○	○
It is hard to know whom to trust when it comes to COVID-19 vaccine information.	○	○	○
I have previously had a negative experience with other vaccines.	○	○	○
I trust the healthcare experts/providers/system.	○	○	○
I have personally experienced mistreatment in the medical care system in the past.	○	○	○
Historical mistreatment of Black people in the medical care system makes me concerned about the COVID-19 vaccines.	○	○	○
My family/friends/co-workers have told me to receive a COVID-19 vaccine.	○	○	○
My family and friends do not plan to get a COVID-19 vaccine.	○	○	○

My place of work/school/institution has told me to receive a COVID-19 vaccine.	○	○	○
A source that I trust (i.e. news, website, etc.) told me to receive a COVID-19 vaccine.	○	○	○
I believe it is better for my family, friends, and community if I receive a COVID-19 vaccine.	○	○	○
I do not know where to sign up for a COVID-19 vaccine.	○	○	○
It will be difficult to travel to a COVID-19 vaccination site.	○	○	○
I do not have health insurance to pay for a COVID-19 vaccine.	○	○	○
I am worried about taking time off from my work/schedule to get a COVID-19 vaccine.	○	○	○
I am worried about missing work if the side effects of a COVID-19 vaccine make me sick.	○	○	○
I can find care for my dependent/child so that I can receive a COVID-19 vaccine.	○	○	○
I am hesitant to get a COVID-19 vaccine because of my religious beliefs.	○	○	○

Vacpt **The following ask about your opinion on recommending the COVID-19 vaccine to a patient.**

Vacpt1 Given what you currently know, how likely would you recommend a COVID-19 vaccine when it becomes available to the patients you consult or treat?

- ○ Extremely likely
- ○ Very likely
- ○ Somewhat likely
- ○ Not too likely
- ○ Not at all likely

Vacpt2 Please **rank** the most important factor for recommending a COVID-19 vaccine to a patient. 1 being **most** important and 7 being **least** important. ***To rank the listed items, drag and drop each item.***

______ Efficacy of vaccine
______ Safety of vaccine and long-term follow up
______ Duration of protection by vaccine
______ Incidence of major and minor adverse effects
______ Recommendation of vaccine by political officials
______ Recommendation of vaccine by healthcare authorities
______ Current exposure to patients with active COVID-19 infection and risk of virus spread
